# Supplementary material for: Heterosis Is a Systemic Property Emerging From Non-linear Genotype-Phenotype Relationships: Evidence From in Vitro Genetics and Computer Simulations
Source: Front Genet. 2018 May 15;9:159. doi: 10.3389/fgene.2018.00159 (PMC5968397; doi:10.3389/fgene.2018.00159)
Supplement: Supplementary file 1 [file Data_Sheet_1.zip › Fievet_et_al_Supp_data.pdf]

Heterosis is a systemic property emerging from nonlinear genotype-phenotype relationships: evidence from *in vitro* genetics and computer simulations

Julie B. Fiévet, Thibault Nidelet, Christine Dillmann, Dominique de Vienne

Supporting information

Supporting Information Legends

S1 Appendix Constrained E_{tot}: consequence on the c_{v}'s of enzyme concentrations.

S2 Appendix Comparing initial and actual c_{v}'s of enzyme concentrations in the glycolysis/fermentation system.

S1 Table Enzyme concentrations (mg.L^{-1}) of the parents of the 61 *in vitro* crosses.

S2 Table Flux values (μM.s^{-1}) in parent and hybrid replicates.

S3 Table Parameters of the variable enzymes.

S4 Table Percentages of the four types of inheritance for the three fluxes in different networks.

S1 Fig. Heterosis types and threshold values of heterosis indices.

S2 Fig. Flux responses to variations of enzyme concentration when E_{tot} is free.

S3 Fig. Flux responses to variations of enzyme concentration when E_{tot} is fixed.

S4 Fig. Comparison of the percentages of different types of inheritance with and without convexity in the enzyme-flux relationship.

S5 Fig. Relationship between parental (J_1 and J_2) and hybrid fluxes.

S6 Fig. Examples of relationship between inheritance and enzyme concentrations.

S7 Fig. Relationship between heterosis and the position of the parents in the concentration space of two enzymes.

S8 Fig. The geometry of heterosis for a constant enzymatic distance between parents, with constraint on E_{tot}.

S9 Fig. Deviations from the means and c_{v}'s of enzyme concentrations relative to the initial values used in the simulations of the glycolysis/fermentation system.

S1 Appendix Constrained $E_{\rm tot}$: consequence on the $c_{\rm v}$'s of enzyme concentrations. Consider the concentration X_j of enzyme j before constraint on $E_{\rm tot}$. Its expectation is $\mathrm{E}(X_j) = \mu_j$ and its variance:

$$\mathrm{Var}(X_j) = (c_{\rm v}\mu_j)^2. \tag{1}$$

Consider now the variable $Y = \sum_j X_j$. Its expectation is:

$$\mathrm{E}(Y) = \sum_j \mu_j = \mu_Y$$

and its variance:

$$\mathrm{Var}(Y) = c_{\rm v}^2 \sum_j \mu_j^2.$$

The variable associated with the concentration of enzyme i upon constraint on $E_{\rm tot}$ is $Z_i = X_i/Y$. Because Y includes X_i, these two variables are not independent. Using the Delta method (Armitage et al., 2002), we can write:

$$\mathrm{E}(Z_i) \approx \frac{\mu_i}{\mu_Y}$$

and

$$\mathrm{Var}(Z_i) \approx \frac{c_{\rm v}^2\mu_i^2}{\mu_Y^4}\left(\mu_Y^2 + \sum_j \mu_j^2 - 2\mu_i\mu_Y\right).$$

Using Eq 1, we get:

$$\frac{\mathrm{Var}(Z_i)}{\mathrm{Var}(X_i)} \approx \frac{\mu_Y^2 + \sum_j \mu_j^2 - 2\mu_i\mu_Y}{\mu_Y^4}.$$

We see that:

$$\forall i, \frac{\mathrm{Var}(Z_i)}{\mathrm{Var}(X_i)} < 1,$$

which means that the constraint decreases the variance of every enzyme concentration. Regarding the coefficient of variation c_i of Z_i, we have:

$$c_i \approx \frac{c_{\rm v}}{\mu_Y}\sqrt{\mu_Y^2 + \sum_j \mu_j^2 - 2\mu_i\mu_Y}. \tag{2}$$

Thus $c_i/c_{\rm v} = 1$ if $\mu_i = \sum_j \mu_j^2/2\mu_Y$, otherwise $c_i/c_{\rm v} < 1$ (resp. $c_i/c_{\rm v} > 1$) when $\mu_i > \sum_j \mu_j^2/2\mu_Y$ (resp. $\mu_i < \sum_j \mu_j^2/2\mu_Y$). In other words the constraint changes the coefficients of variation in an inverse relation to the enzyme concentrations: the coefficient of variation of the most (resp. less) abundant enzymes will decrease (resp. increase) under constrained $E_{\rm tot}$. This theoretical prediction is consistent with observations. For instance, when $c_{\rm v} = 0.6$ in the four-enzyme system, the theoretical coefficients of variation after constraint were 0.63, 0.58, 0.30 and 0.71, for PGI, PFK, FBA and TPI, respectively, and we observed 0.61, 0.54, 0.31 and 0.76. The mean was 0.56, which is close to 0.6.

S2 Appendix Comparing initial and actual $c_{\rm v}$'s of enzyme concentrations in the glycolysis/fermentation system. In our simulations of the

glycolysis/fermentation network, the steady state was not necessarily reached, and depended on enzyme concentration values. If one member of the triplet parents-hybrid did not reach a steady state, the triplet was excluded, which was all the more frequent when c_v's were high. The following table shows the percentage of successful simulations under different c_v values, without and with contraint on E_tot.

c_v	0.1	0.2	0.3	0.4	0.5	0.6	0.7
Free E_tot	95.8	77.5	63.4	51.9	40.3	33.2	28.1
Fixed E_tot	99.3	86.4	69.3	53.9	41.3	32.9	27.6

Once 10 000 successful simulations were obtained for each condition, we examined the histograms of glucose flux and observed that there were null values, particularly for the highest c_v's. The triplets involved were elimitated, so that the total number of simulations finally retained varied according to the c_v and the presence/absence of constraint:

c_v	0.1	0.2	0.3	0.4	0.5	0.6	0.7
Free E_tot	10 000	9 998	9 727	8 634	6 738	4 812	3 162
Fixed E_tot	10 000	10 000	10 000	9 899	9 340	8 202	6 581

Given the high number of excluded simulations for the highest c_v's, we checked for possible biases in the *a posteriori* distributions of enzyme concentrations. This was actually found to be the case, as shown S9 Fig. With free E_tot, the mean concentrations of PFK, FBA and ADH, and to a lesser extent of PGK, were higher than the reference concentrations, while the concentration of HK was lower, this effect being positively related to the initial c_v values (S9 FigA). This means that, frequently, the lowest concentrations of some enzymes (the highest for HK) did not allow the steady state to be reached or resulted in null fluxes, and so the corresponding triplets were excluded. As a consequence, means and variances were modified and c_v's changed accordingly, the c_v of HK being the most reduced for high initial c_v values (S9 FigB).

With fixed E_tot, the *a posteriori* means were markedly different from the reference concentrations for the highest c_v's (S9 Fig C). This is likely due to HK, which is by far the most abundant enzyme (77.5 % of the total concentration): whenever a high HK value was drawn, application of the constraint reduced the concentration of the other enzymes, which can prevent the steady state from being reached or can lead to null fluxes. Therefore the mean concentration of HK was reduced and the concentrations of the other enzymes were increased, the variances of the concentrations being decreased in both cases. The resulting c_v's are shown S9 FigE, in comparison with the theoretical values that are expected in the case of constraint (Eq 2 and S9 FigD): again, discrepancies were observed for the highest c_v's and depended on the enzyme. In these cases, observed c_v's were generally lower than theoretical c_v's.

S1 Table Enzyme concentrations (mg.L^{-1}) of the parents of the 61 *in vitro* crosses. PGI, phosphoglucose isomerase; PFK, phosphofructokinase; FBA, fructose-1,6-bisphosphate aldolase; TPI, triosephosphate isomerase.

Cross	Parent 1					Parent 2				
#	#	PGI	PFK	FBA	TPI	#	PGI	PFK	FBA	TPI
1	24	70	5	5	21.9	40	25	70	2	4.9
2	30	40	35	5	21.9	38	35	60	5	1.9
3	27	55	15	12	19.9	38	35	60	5	1.9
4	25	20	10	5	66.9	36	25	50	12	14.9
5	27	55	15	12	19.9	35	15	50	5	31.9
6	29	10	20	15	56.9	30	40	35	5	21.9
7	24	70	5	5	21.9	26	40	10	40	11.9
8	14	4.23	2.62	76.42	18.62	16	3.72	1.95	86.61	9.61
9	16	3.72	1.95	86.61	9.61	13	5.79	3.3	76.42	16.38
10	15	9.4	2.58	86.61	3.31	23	3.4	2.81	86.61	9.08
11	14	4.23	2.62	76.42	18.62	23	3.4	2.81	86.61	9.08
12	23	3.4	2.81	86.61	9.08	17	7.36	3.21	86.61	4.72
13	17	7.36	3.21	86.61	4.72	22	9.88	3.73	86.61	1.67
14	40	25	70	2	4.9	28	15	15	55	16.9
15	1	39.32	12.38	25.47	24.71	2	51.97	14.22	25.47	10.23
16	15	9.4	2.58	86.61	3.31	8	3.33	5.75	66.23	26.58
17	23	3.4	2.81	86.61	9.08	12	5.92	5.25	76.42	14.3
18	13	5.79	3.3	76.42	16.38	8	3.33	5.75	66.23	26.58
19	32	15	40	45	1.9	37	25	50	20	6.9
20	13	5.79	3.3	76.42	16.38	19	29.71	6.62	56.04	9.53
21	17	7.36	3.21	86.61	4.72	8	3.33	5.75	66.23	26.58
22	14	4.23	2.62	76.42	18.62	20	13.29	8.21	56.04	24.36
23	17	7.36	3.21	86.61	4.72	19	29.71	6.62	56.04	9.53
24	2	51.97	14.22	25.47	10.23	5	15.66	23.52	45.85	16.86
25	15	9.4	2.58	86.61	3.31	18	37.9	12.6	35.66	15.73
26	15	9.4	2.58	86.61	3.31	1	39.32	12.38	25.47	24.71
27	22	9.88	3.73	86.61	1.67	21	20.6	7.18	66.23	7.89
28	14	4.23	2.62	76.42	18.62	2	51.97	14.22	25.47	10.23
29	18	37.9	12.6	35.66	15.73	7	6.79	20.43	56.04	18.63
30	13	5.79	3.3	76.42	16.38	1	39.32	12.38	25.47	24.71
31	2	51.97	14.22	25.47	10.23	8	3.33	5.75	66.23	26.58
32	19	29.71	6.62	56.04	9.53	18	37.9	12.6	35.66	15.73
33	12	5.92	5.25	76.42	14.3	1	39.32	12.38	25.47	24.71
34	19	29.71	6.62	56.04	9.53	20	13.29	8.21	56.04	24.36
35	10	4.24	25.75	66.23	5.67	4	32.45	28.74	35.66	5.03
36	11	8.44	9.53	66.23	17.69	4	32.45	28.74	35.66	5.03
37	13	5.79	3.3	76.42	16.38	4	32.45	28.74	35.66	5.03
38	20	13.29	8.21	56.04	24.36	8	3.33	5.75	66.23	26.58
39	11	8.44	9.53	66.23	17.69	6	15.55	30.53	45.85	9.96
40	20	13.29	8.21	56.04	24.36	9	9.01	8.8	66.23	17.85
41	5	15.66	23.52	45.85	16.86	4	32.45	28.74	35.66	5.03
42	10	4.24	25.75	66.23	5.67	6	15.55	30.53	45.85	9.96

Continued on next page

Continued from the previous page										
**Cross**	**Parent 1**					**Parent 2**				
#	#	PGI	PFK	FBA	TPI	#	PGI	PFK	FBA	TPI
43	7	6.79	20.43	56.04	18.63	6	15.55	30.53	45.85	9.96
44	16	3.72	1.95	86.61	9.61	7	6.79	20.43	56.04	18.63
45	12	5.92	5.25	76.42	14.3	4	32.45	28.74	35.66	5.03
46	18	37.9	12.6	35.66	15.73	9	9.01	8.8	66.23	17.85
47	5	15.66	23.52	45.85	16.86	7	6.79	20.43	56.04	18.63
48	15	9.4	2.58	86.61	3.31	10	4.24	25.75	66.23	5.67
49	12	5.92	5.25	76.42	14.3	3	12.2	27.64	35.66	26.39
50	11	8.44	9.53	66.23	17.69	2	51.97	14.22	25.47	10.23
51	16	3.72	1.95	86.61	9.61	6	15.55	30.53	45.85	9.96
52	3	12.2	27.64	35.66	26.39	9	9.01	8.8	66.23	17.85
53	5	15.66	23.52	45.85	16.86	9	9.01	8.8	66.23	17.85
54	17	7.36	3.21	86.61	4.72	1	39.32	12.38	25.47	24.71
55	21	20.6	7.18	66.23	7.89	6	15.55	30.53	45.85	9.96
56	21	20.6	7.18	66.23	7.89	3	12.2	27.64	35.66	26.39
57	21	20.6	7.18	66.23	7.89	5	15.66	23.52	45.85	16.86
58	10	4.24	25.75	66.23	5.67	22	9.88	3.73	86.61	1.67
59	10	4.24	25.75	66.23	5.67	12	5.92	5.25	76.42	14.3
60	22	9.88	3.73	86.61	1.67	3	12.2	27.64	35.66	26.39
61	17	7.36	3.21	86.61	4.72	5	15.66	23.52	45.85	16.86

S2 Table Flux values (μM.s^{-1}) in parent and hybrid replicates.

Cross	Parent 1			Parent 2			Hybrid		
1	1.19	1.31	1.15	0.68	0.8	-	1.08	1.13	1.1
2	1.74	1.7	1.73	1.88	1.89	-	1.64	1.66	1.56
3	4.53	4.52	4.62	1.88	1.89	-	2.38	2.32	2.26
4	1.88	1.83	1.9	4.04	4.15	4.34	2.45	2.32	2.25
5	4.53	4.52	4.62	1.79	1.79	1.79	2.16	2.45	2.56
6	5.27	4.86	5.33	1.74	1.7	1.73	2.56	2.43	2.49
7	1.19	1.31	1.15	10.3	9.57	9.22	5.28	4.14	4.18
8	5.57	5.38	5.42	3.89	4.14	3.94	4.56	4.68	4.64
9	3.89	4.14	3.94	6.52	6.04	6.57	5.08	5.34	4.96
10	5.06	5.18	4.92	6.97	6.66	7.14	6.02	6.02	6.29
11	5.57	5.38	5.42	6.97	6.66	7.14	6.15	5.98	6.22
12	6.97	6.66	7.14	6.39	6.46	6.55	6.59	6.49	6.41
13	6.39	6.46	6.55	7.33	7.34	7.26	7.35	7.28	7.13
14	0.68	0.8	-	12.3	13.1	13.3	8.3	7.07	7.33
15	8.5	8.34	8.55	8.69	8.23	8.43	7.8	7.28	7.86
16	5.06	5.18	4.92	9.19	9.27	9.25	8.1	7.52	7.5
17	6.97	6.66	7.14	8.97	8.86	8.87	7.63	7.84	7.66
18	6.52	6.04	6.57	9.19	9.27	9.25	7.88	8.04	7.83
19	11.5	11.7	12	6.75	6.55	6.63	8.51	8.05	-
20	6.52	6.04	6.57	9.64	9.3	9.24	8.48	8.19	8.4
21	6.39	6.46	6.55	9.19	9.27	9.25	8.59	8.42	8.48
22	5.57	5.38	5.42	10.03	10.08	10.13	9.05	8.89	8.96
23	6.39	6.46	6.55	9.64	9.3	9.24	8.69	9.26	9.31
24	8.69	8.23	8.43	12.29	11.9	12.25	9.71	9.39	9.4
25	5.06	5.18	4.92	9.69	9.79	9.76	9.92	9.65	9.58
26	5.06	5.18	4.92	8.5	8.34	8.55	9.83	9.75	9.61
27	7.33	7.34	7.26	11.59	11.54	11.43	9.74	9.8	9.75
28	5.57	5.38	5.42	8.69	8.23	8.43	9.91	9.77	9.64
29	9.69	9.79	9.76	11.65	11.37	11.64	10.09	9.74	9.56
30	6.52	6.04	6.57	8.5	8.34	8.55	9.91	9.85	9.82
31	8.69	8.23	8.43	9.19	9.27	9.25	10.13	10.05	9.96
32	9.64	9.3	9.24	9.69	9.79	9.76	10.22	10.27	10.29
33	8.97	8.86	8.87	8.5	8.34	8.55	10.43	10.28	10.41
34	9.64	9.3	9.24	10.03	10.08	10.13	10.5	10.46	10.6
35	11.39	11.71	11.99	9.83	9.65	9.56	10.76	10.66	10.22
36	11.31	11.22	11.12	9.83	9.65	9.56	10.77	10.84	10.08
37	6.52	6.04	6.57	9.83	9.65	9.56	10.49	10.83	10.81
38	10.03	10.08	10.13	9.19	9.27	9.25	10.57	10.67	11
39	11.31	11.22	11.12	10.63	10.39	10.19	10.97	10.87	10.52
40	10.03	10.08	10.13	11.66	11.21	11.29	10.96	10.68	-
41	12.29	11.9	12.25	9.83	9.65	9.56	11.23	10.69	10.72
42	11.39	11.71	11.99	10.63	10.39	10.19	11.21	10.95	10.69
43	11.65	11.37	11.64	10.63	10.39	10.19	10.84	11.15	11.04
44	3.89	4.14	3.94	11.65	11.37	11.64	10.86	11.17	11.05

Continued on next page

Continued from the previous page									
**Cross**	**Parent 1**			**Parent 2**			**Hybrid**		
45	8.97	8.86	8.87	9.83	9.65	9.56	11.38	10.94	10.82
46	9.69	9.79	9.76	11.66	11.21	11.29	11.29	11.15	10.8
47	12.29	11.9	12.25	11.65	11.37	11.64	11.03	11.18	11.13
48	5.06	5.18	4.92	11.39	11.71	11.99	11.6	11.06	-
49	8.97	8.86	8.87	10.95	11.22	11.39	11.59	11.49	11.39
50	11.31	11.22	11.12	8.69	8.23	8.43	11.48	11.52	11.62
51	3.89	4.14	3.94	10.63	10.39	10.19	11.42	11.43	11.83
52	10.95	11.22	11.39	11.66	11.21	11.29	11.27	11.84	11.6
53	12.29	11.9	12.25	11.66	11.21	11.29	11.55	11.53	11.64
54	6.39	6.46	6.55	8.5	8.34	8.55	11.79	11.54	11.55
55	11.59	11.54	11.43	10.63	10.39	10.19	11.67	11.58	11.68
56	11.59	11.54	11.43	10.95	11.22	11.39	11.65	11.77	11.84
57	11.59	11.54	11.43	12.29	11.9	12.25	11.73	11.85	-
58	11.39	11.71	11.99	7.33	7.34	7.26	12.22	12.37	12.11
59	11.39	11.71	11.99	8.97	8.86	8.87	12.43	12.19	12.23
60	7.33	7.34	7.26	10.95	11.22	11.39	12.32	12.42	12.66
61	6.39	6.46	6.55	12.29	11.9	12.25	12.79	12.59	12.5

S3 Table Parameters of the variable enzymes. $V_{\max}$: maximal velocity, k_{cat}: catalytic constant, Mr: molecular mass, E_{ref}: reference concentration. For the simulations, we used $E_{\text{ref}}/5$ (see Materials and methods).

**Enzyme**	$\boldsymbol{V_{\max}}$ mMol.min^{-1}	$\boldsymbol{k_{\text{cat}}}$ min^{-1}	**Mr** g/mMol	$\boldsymbol{E_{\text{ref}}}$ mg
HK (E.C. 2.7.1.1)	236.70	105.6	55	123281.25
PGI (E.C. 5.3.1.9)	1056.00	84600.0	120	1466.67
PFK (E.C. 2.7.1.11)	110.00	554400.0	790	156.75
PGK (E.C. 2.7.2.3)	1288.00	21240.0	50	3032.02
FBA (E.C. 4.1.2.13)	94.69	12500.0	80	606.02
PGM (E.C. 5.4.2.11)	2585.00	29400.0	112.4	9882.79
ENO (E.C. 4.2.1.11)	201.60	4680.0	90	3876.92
PYK (E.C. 2.7.1.40)	1000.00	13920.0	216	15517.24
PDC (E.C. 4.1.1.1)	857.80	232000.0	250	924.35
ADH (E.C. 1.1.1.1)	209.50	47166.0	60	266.51
G3*P*DH (E.C. 1.1.1.8)	47.11	20700.0	70	159.31

S4 Table Percentages of the four types of inheritance for the three fluxes in different networks. A: Normal network. B: With constant hexokinase. C: No glycerol branch. D: With constant hexokinase and no glycerol branch (see S4 Fig).

**Constraint**		**Free $\boldsymbol{E_{\text{tot}}}$**				**Fixed $\boldsymbol{E_{\text{tot}}}$**			
**Network**		A	B	C	D	A	B	C	D
**Glucose**	BPH	23.25	33.93	21.54	3.48	35.58	34.46	30.95	3.78
	+MPH	56.12	64.98	38.09	96.52	58.60	64.44	46.87	96.22
	–MPH	20.64	1.09	40.38	0.00	5.82	1.10	22.19	0.00
	WPH	0.00	0.00	0.00	0.00	0.00	0.00	0.00	0.00
**Glycerol**	BPH	16.94	16.02	0	0	20.07	15.14	0	0
	+MPH	35.12	45.37	0	0	33.10	46.51	0	0
	–MPH	47.01	28.86	0	0	43.98	27.69	0	0
	WPH	0.93	9.75	0	0	2.84	10.66	0	0
**Acetaldehyde**	BPH	24.88	33.73	21.54	3.48	36.83	34.76	30.95	3.78
	+MPH	55.79	64.48	38.09	96.52	56.40	62.75	46.77	96.22
	–MPH	19.32	1.79	40.38	0.00	6.77	2.49	22.29	0.00
	WPH	0.01	0.00	0.00	0.00	0.00	0.00	0.00	0.00

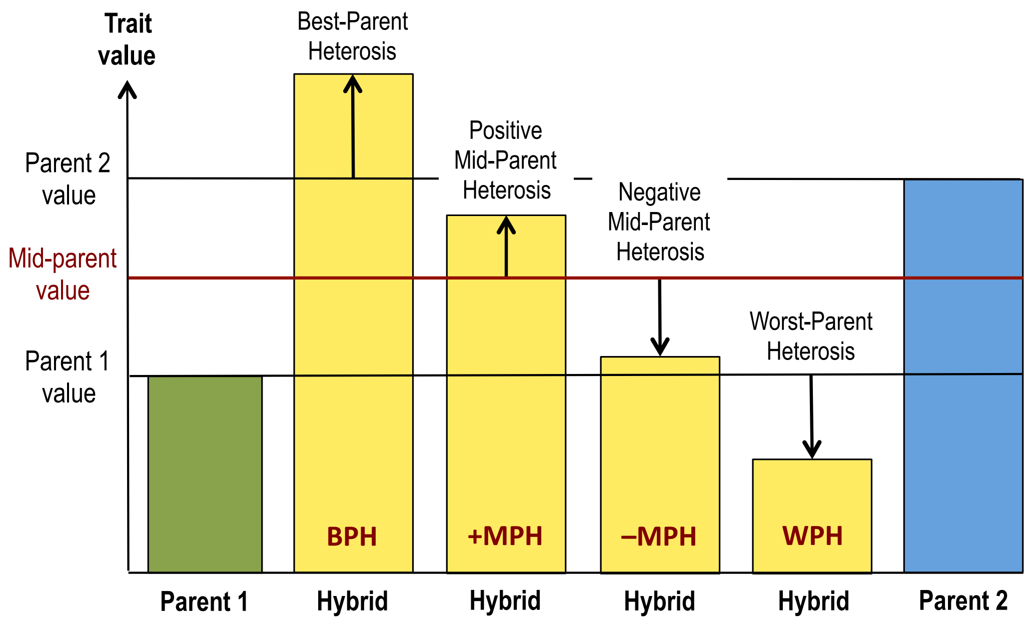

Heterosis index	BPH	+MPH	–MPH	WPH
H_{PR}	> 1	$0 < H_{\mathrm{PR}} \leq 1$	$-1 \leq H_{\mathrm{PR}} < 0$	< -1
H_{BP}	> 0	≤ 0	≤ 0	≤ 0
H_{WP}	≥ 0	≥ 0	≥ 0	< 0
H_{MP}	> 0	> 0	< 0	< 0

S1 Fig. Heterosis types and threshold values of heterosis indices. H_{PR}: potence ratio. H_{BP}: index of best-parent heterosis. H_{WP}: index of worst-parent heterosis. H_{MP}: index of mid-parent heterosis.

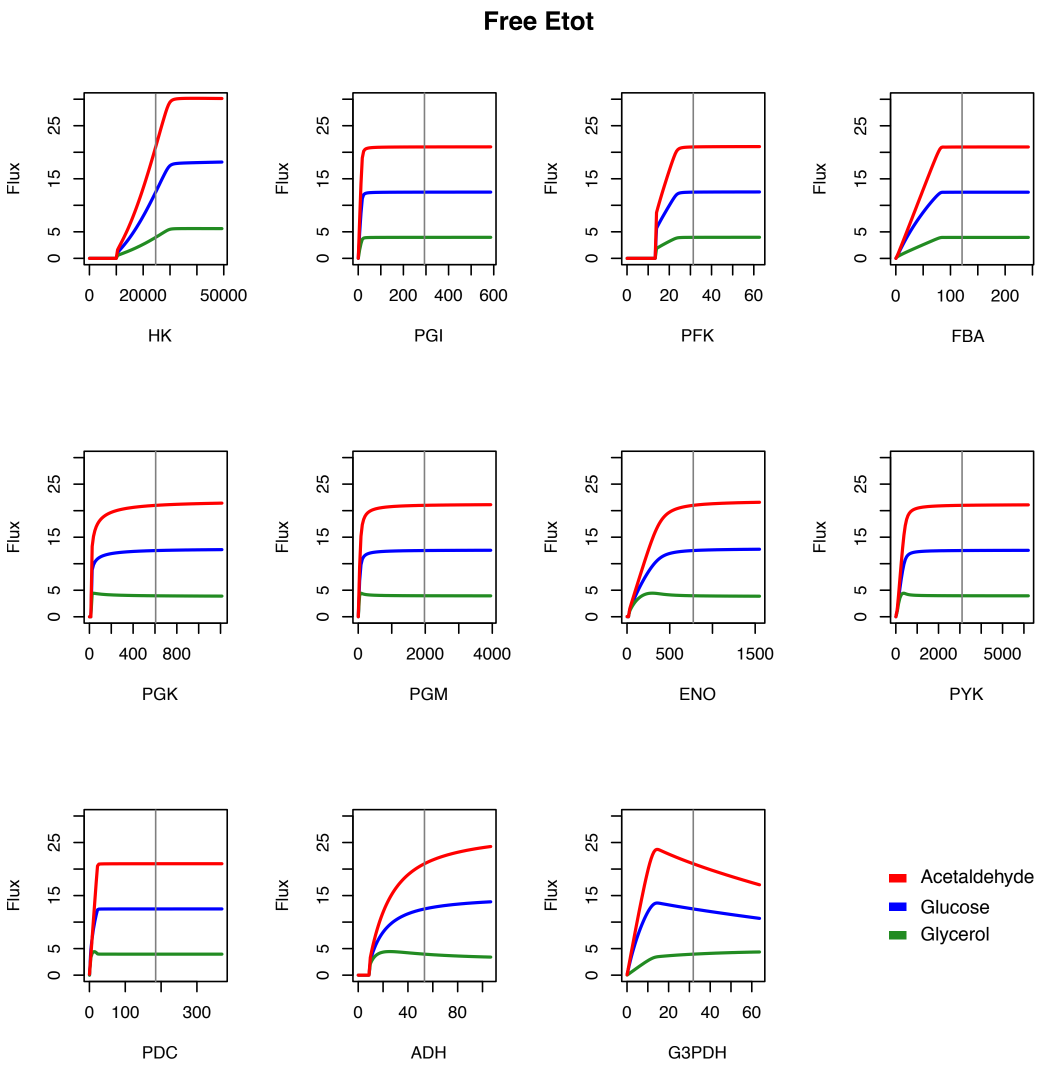

S2 Fig. Flux responses to variations of enzyme concentration when E_{tot} is free. Enzyme concentrations varied from 0 to twice the reference values chosen for the simulations (see S3 Table), with no constraint on E_{tot}. Vertical lines indicate reference concentrations.

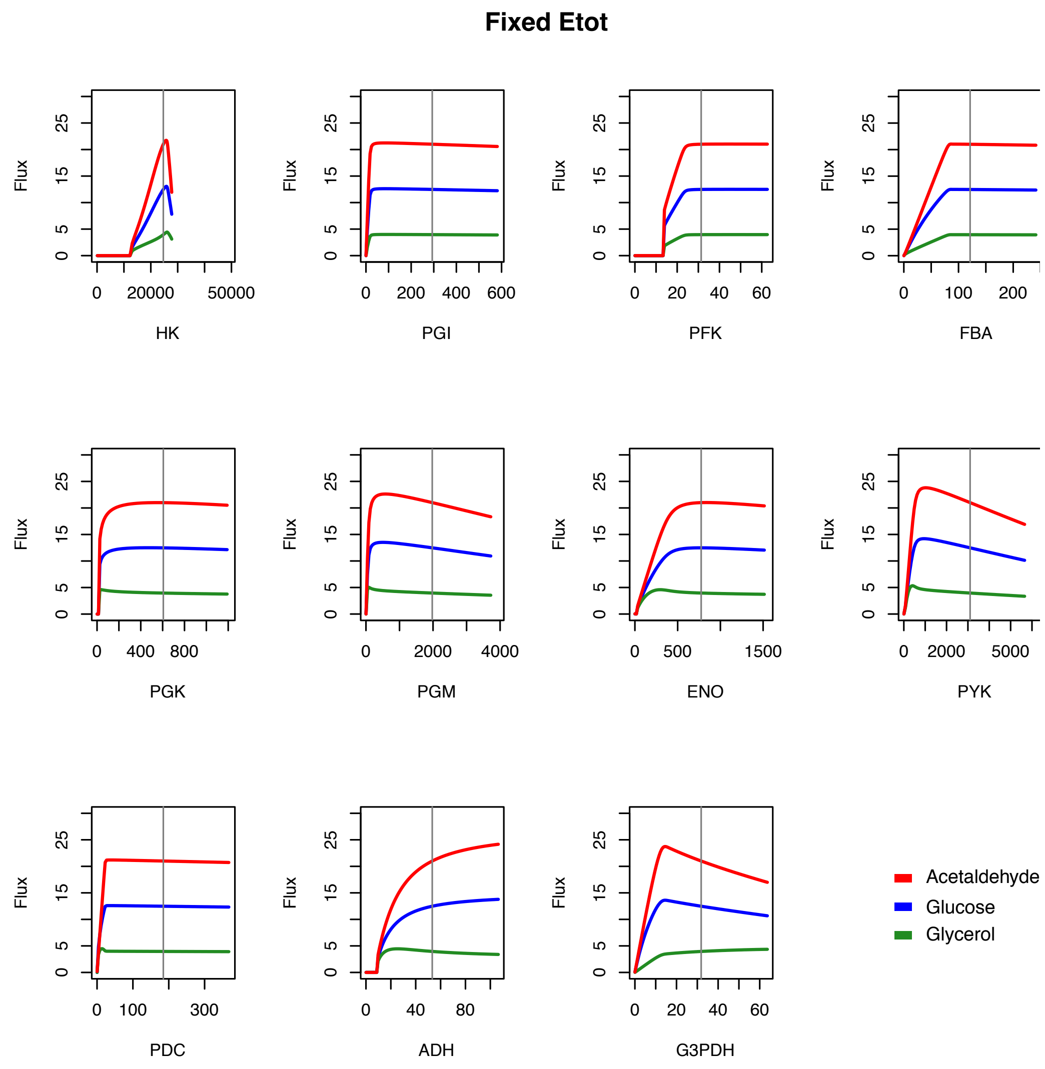

S3 Fig. Flux responses to variations of enzyme concentration when E_{tot} is fixed. Same as S2 Fig, except that E_{tot} was fixed.

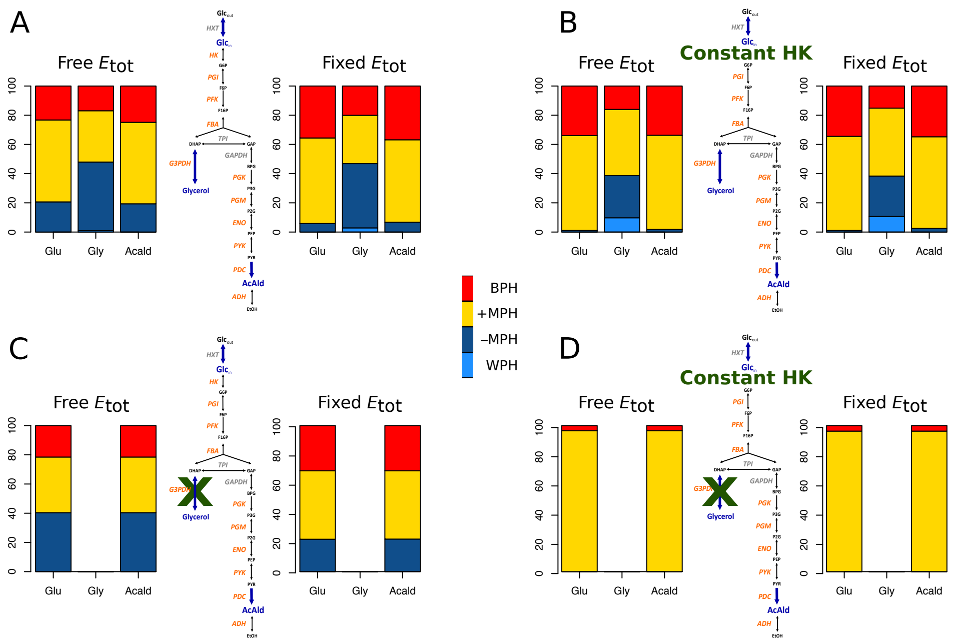

S4 Fig. Comparison of the percentages of different types of inheritance with and without convexity in the enzyme-flux relationship. A: Normal network. B: Network with constant hexokinase. C: Network without the glycerol branch. D: Network with constant hexokinase and without the glycerol branch ($c_v = 0.4$).

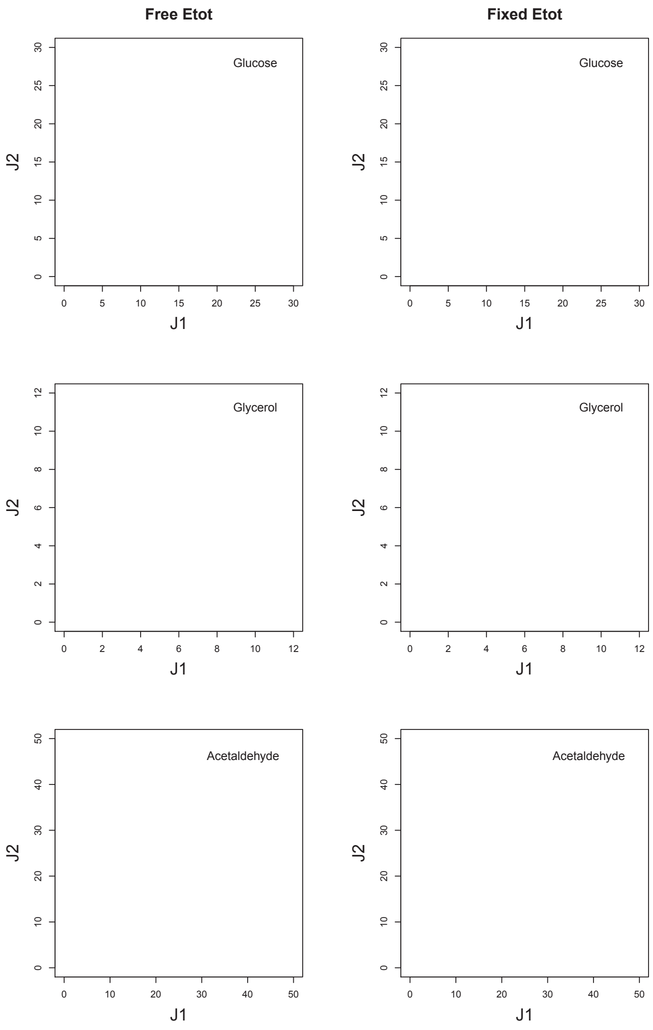

S5 Fig. Relationship between parental (J_1 and J_2) and hybrid fluxes. Symbols as in Fig 8. $c_{\rm v} = 0.4$.

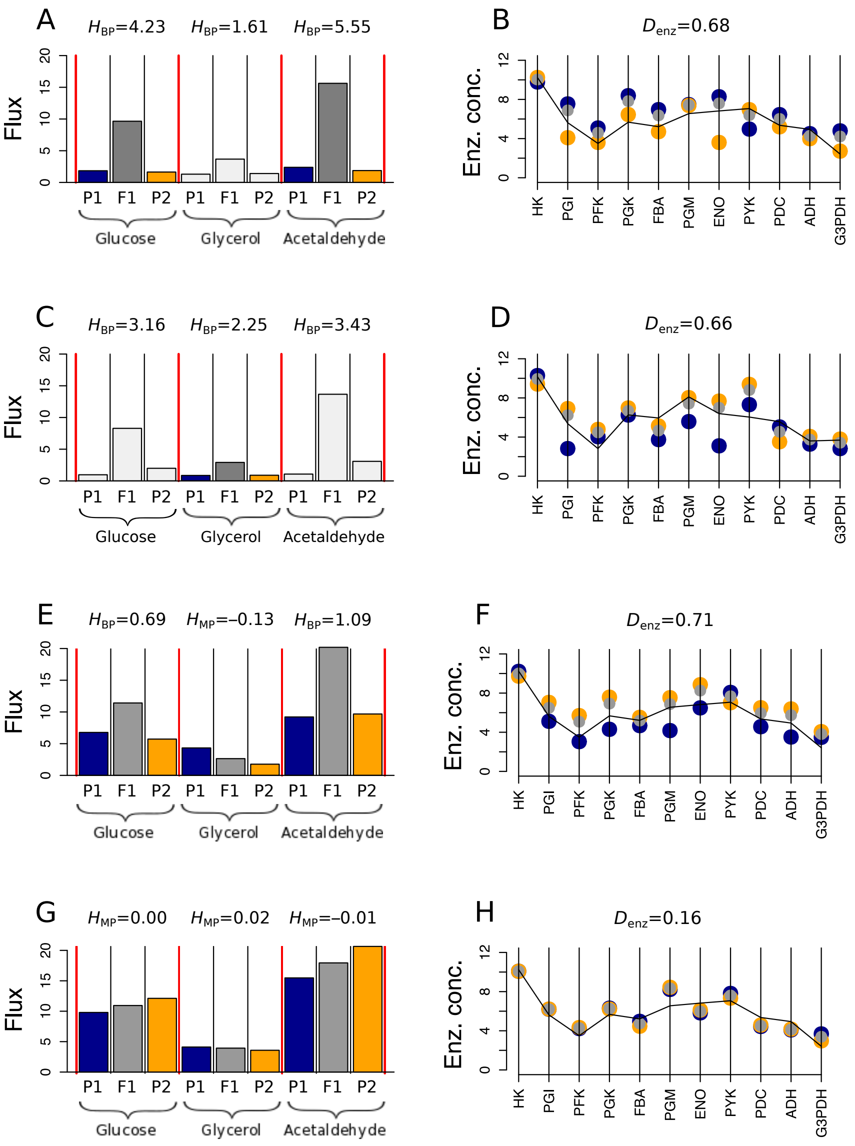

S6 Fig. Examples of relationship between inheritance and enzyme concentrations. ($c_\mathrm{v} = 0.7$, fixed E_tot). A: Parental (blue and orange) and hybrid (gray) fluxes for the cross that displayed the highest heterosis for glucose. This cross also displayed the highest heterosis for acetaldehyde; B: Corresponding enzyme concentrations. C: Fluxes for the cross that displayed the highest heterosis for glycerol. D: Corresponding enzyme concentrations. E: Flux values in the cross between the most distant parents ($D_\mathrm{enz} = 0.71$). F: Corresponding enzyme concentrations. G: Flux values in the cross between the closest parents ($D_\mathrm{enz} = 0.16$). H: Corresponding enzyme concentrations. The broken line in B, D, F and H shows the enzyme concentrations of the parent displaying the highest glucose flux value, as a proxy for the optimal concentration distribution. Enzyme concentrations are log-transformed. When there is no BPH ($H_\mathrm{BP} < 0$) we display the index H_MP, the sign of which indicates whether there is positive or negative MPH.

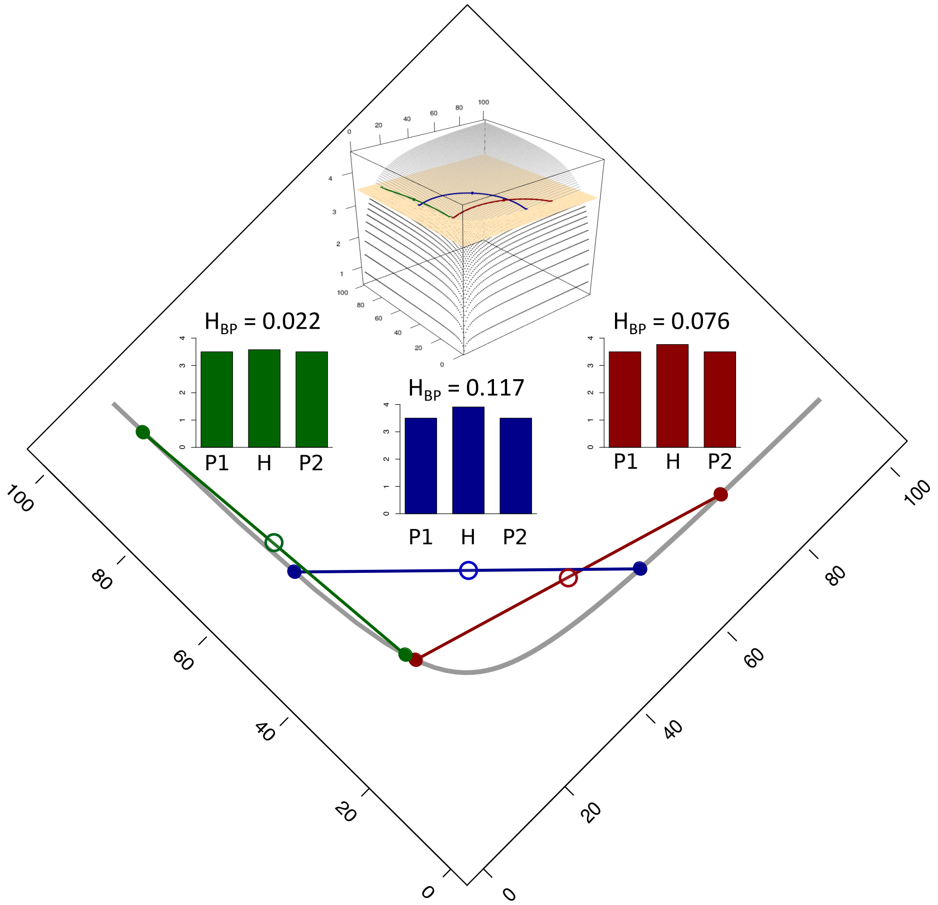

S7 Fig. Relationship between heterosis and the position of the parents in the concentration space of two enzymes. Three pairs of parents (green, blue and red) are on the same level curve (points on the gray curve) defined by the horizontal cutting plane shown on the 3D vignette. D_{enz} is the same for the three pairs of parents. There is additivity of enzyme concentrations in the hybrids (open circles). BPH is higher when parents are complementary for "high" and "low" concentrations (compare blue with red and green cases). The figure is drawn from the equation: $J = 1/(\frac{1}{E_1} + \frac{1}{E_2} + 0.2)$ in arbitrary units. The level curve is at $J = 3.5$, and $D_{\text{enz}} = 60$.

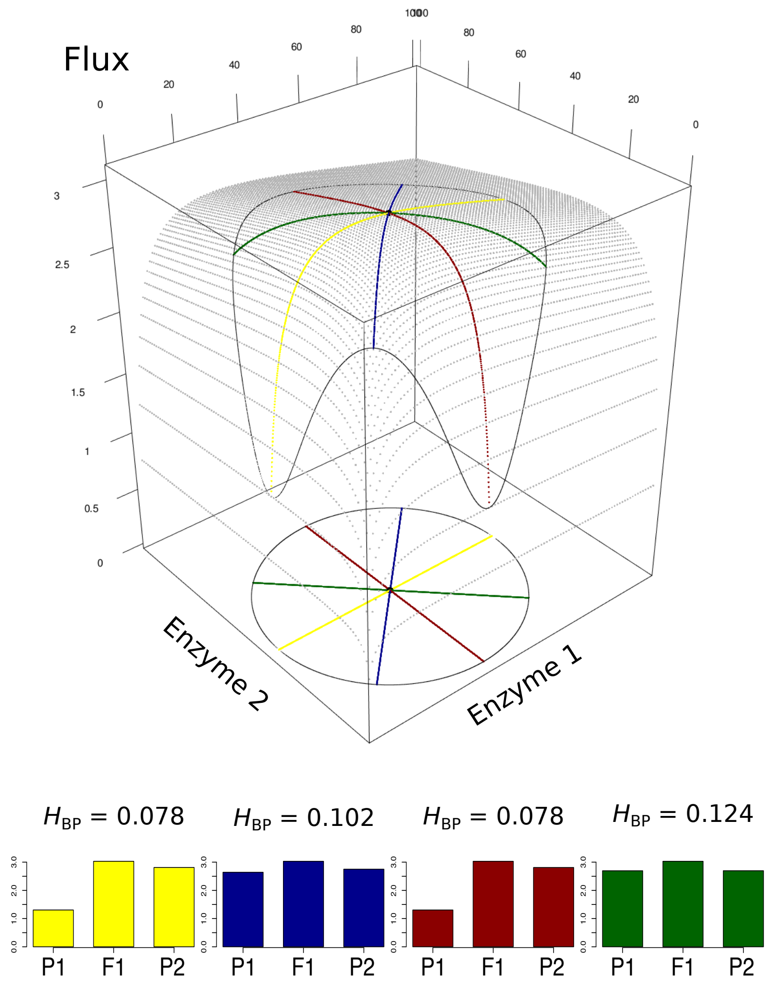

S8 Fig. The geometry of heterosis for a constant enzymatic distance between parents, with constraint on E_{tot}. Parameter values are the same as in Fig 14, but the constraint increases concavity, resulting in BPH in all cases. The flux equation is $J = \frac{1}{\frac{1}{E_1}+\frac{1}{E_2}+0.01(E_1+E2)+0.2}$.

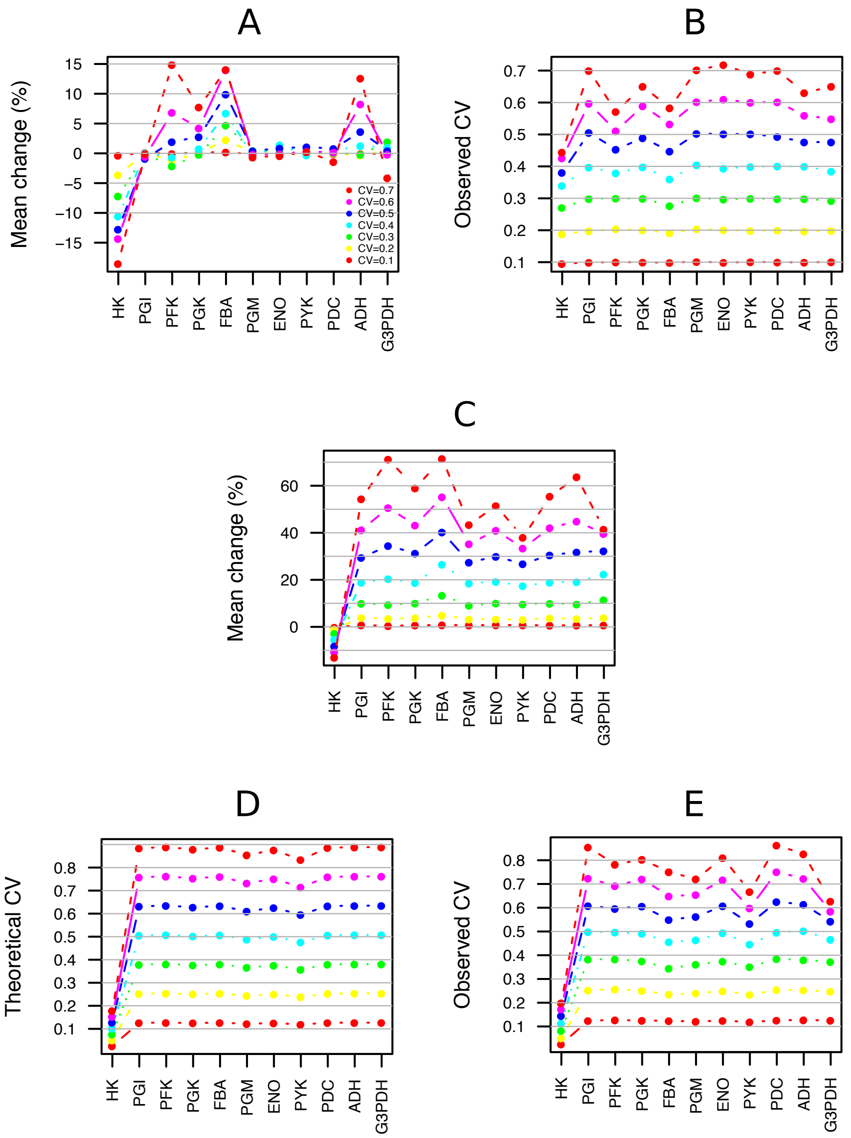

S9 Fig. Deviations from the means and c_v's of enzyme concentrations relative to the initial values used in the simulations of the glycolysis/fermentation system. A and B: Free E_{tot}. A: Variation in the means relative to the reference concentrations of the model (in %). B: Observed c_v's. C, D and E: Fixed E_{tot}. C: Variation in the means relative to the reference concentrations of the model (in %). D: Theoretical c_v's when E_{tot} is constrained; c_v's are inversely proportional to the enzyme concentrations. E: Observed c_v's.

Reference

Armitage. P., Berry, G., and Matthews, J. N. S. (2002). *Statistical methods in medical research*. Malden: Blackwell Publishing,
