# Supplementary figures and images for: Heterosis Is a Systemic Property Emerging From Non-linear Genotype-Phenotype Relationships: Evidence From in Vitro Genetics and Computer Simulations
Source: Front Genet. 2018 May 15;9:159. doi: 10.3389/fgene.2018.00159 (PMC5968397; doi:10.3389/fgene.2018.00159)

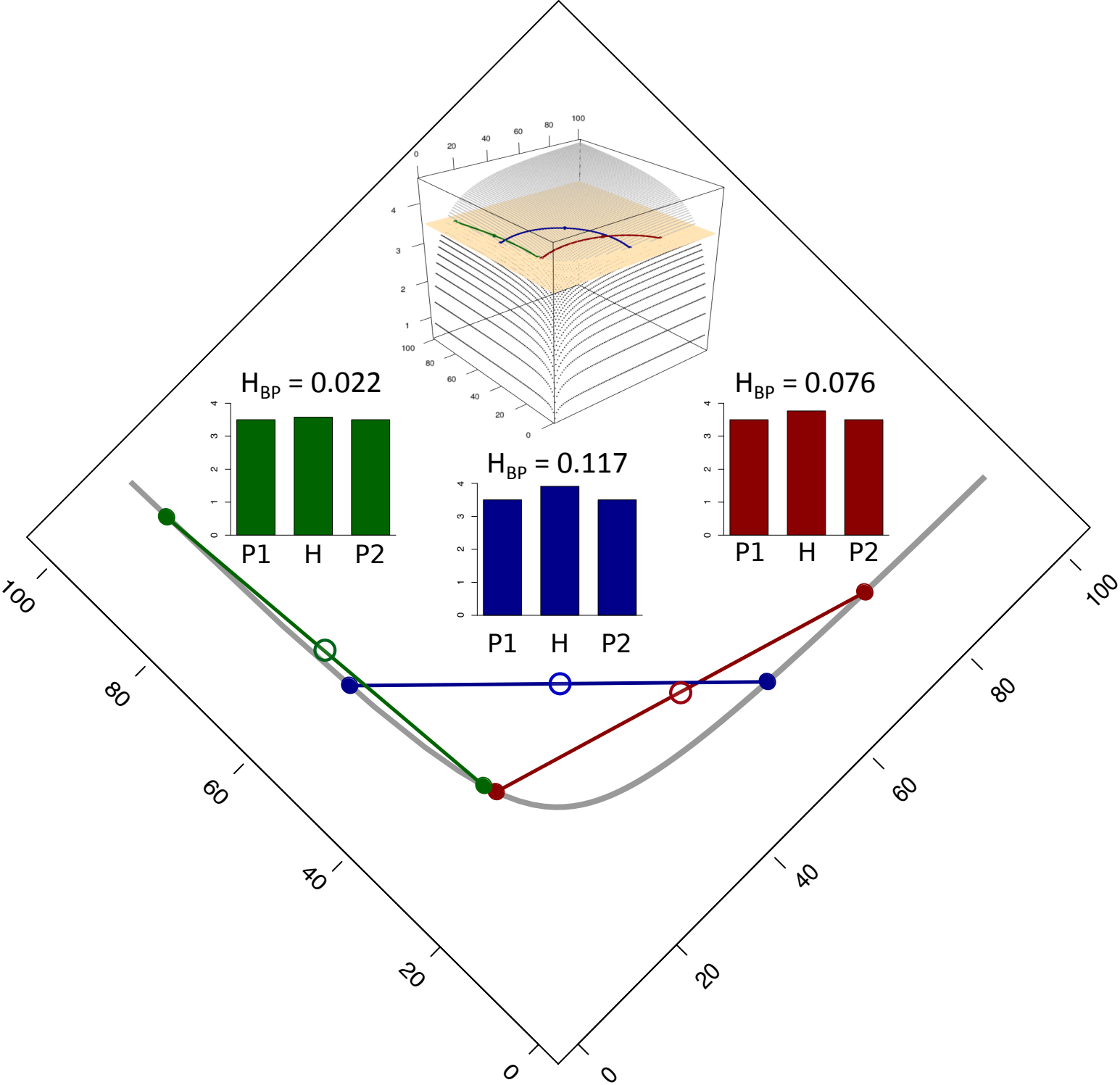

Supplement: Supplementary file 1 [file Data_Sheet_1.zip › Constant_Euclidean_distances.pdf]

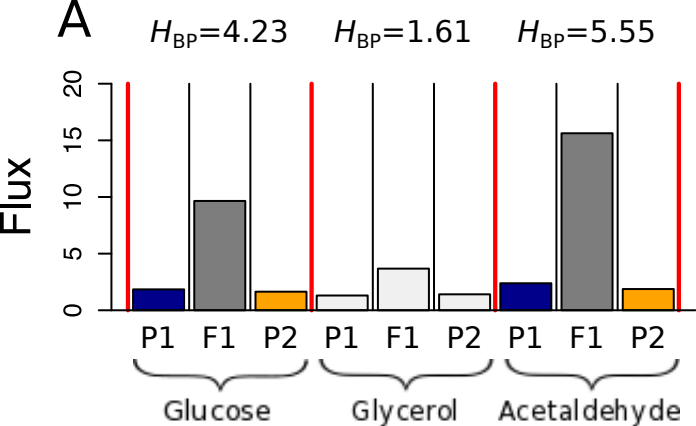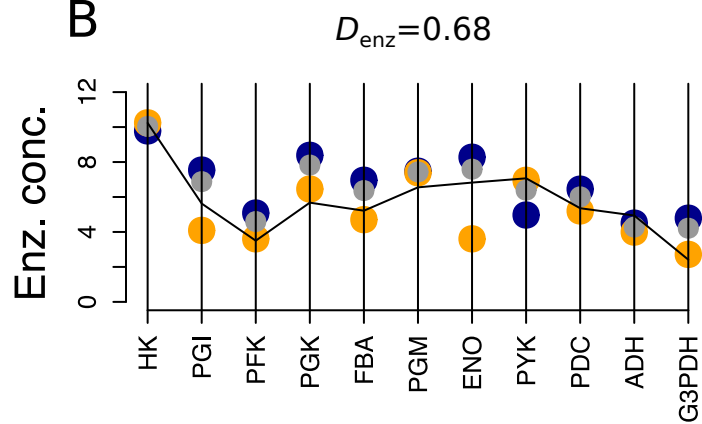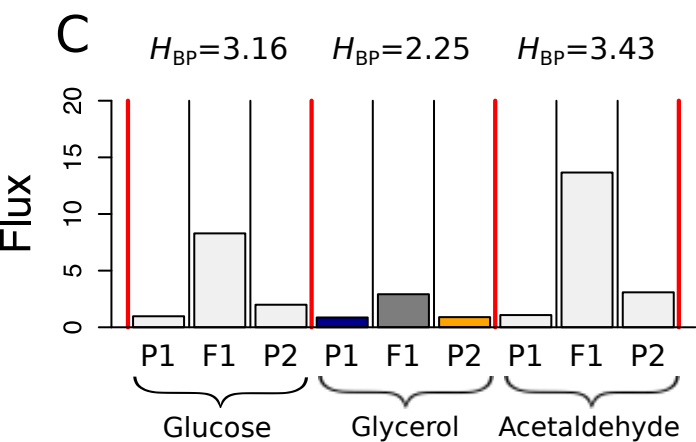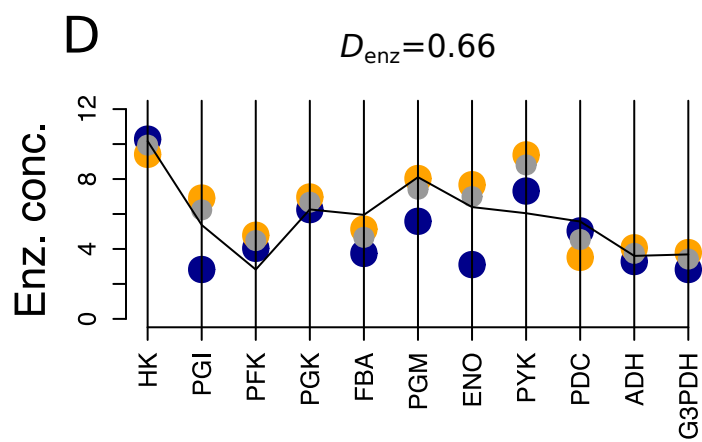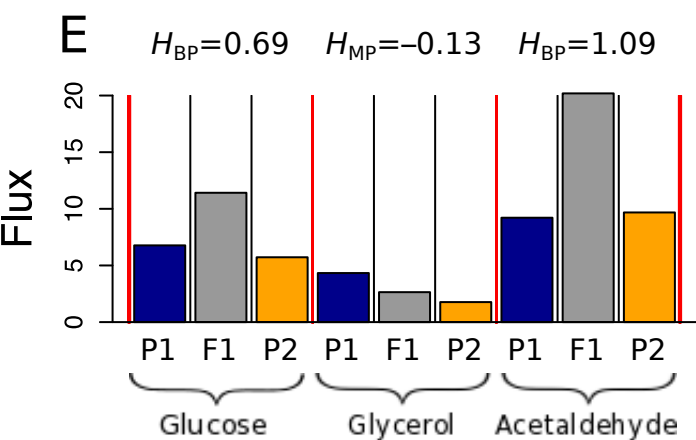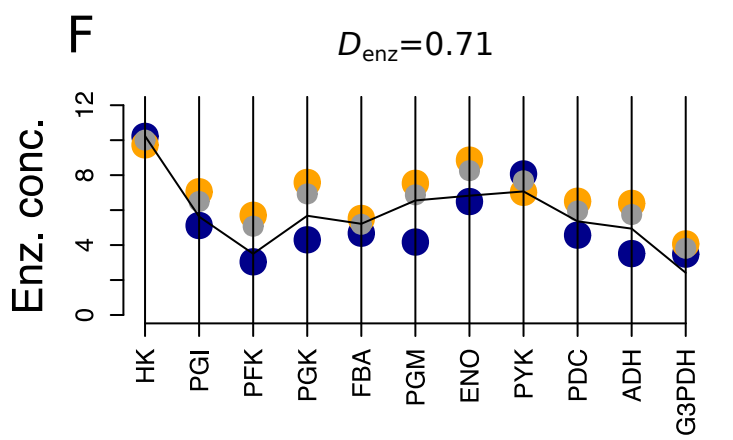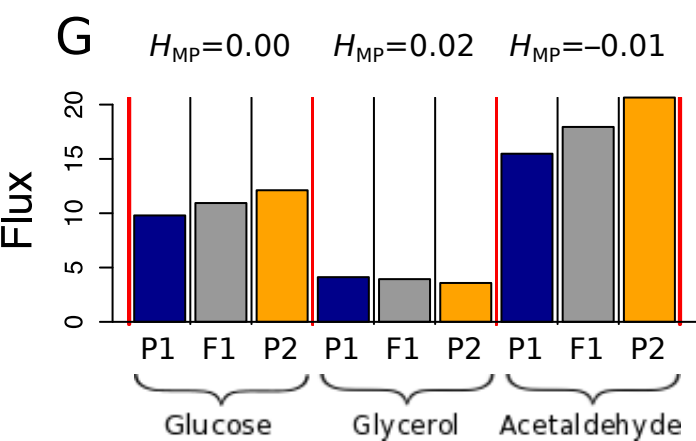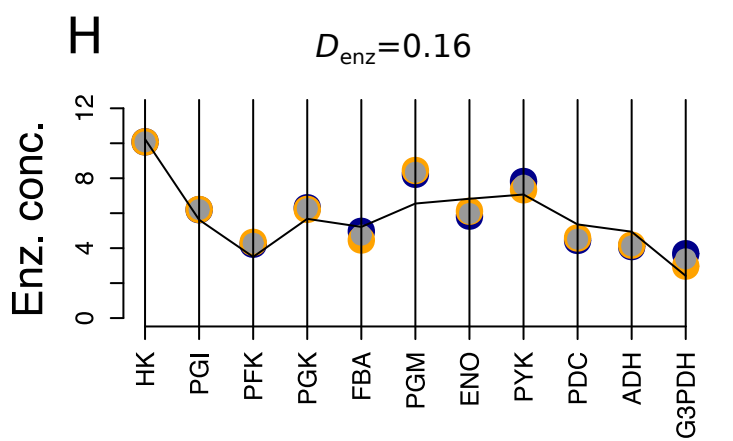

Supplement: Supplementary file 1 [file Data_Sheet_1.zip › Enzyme_distributions.pdf]

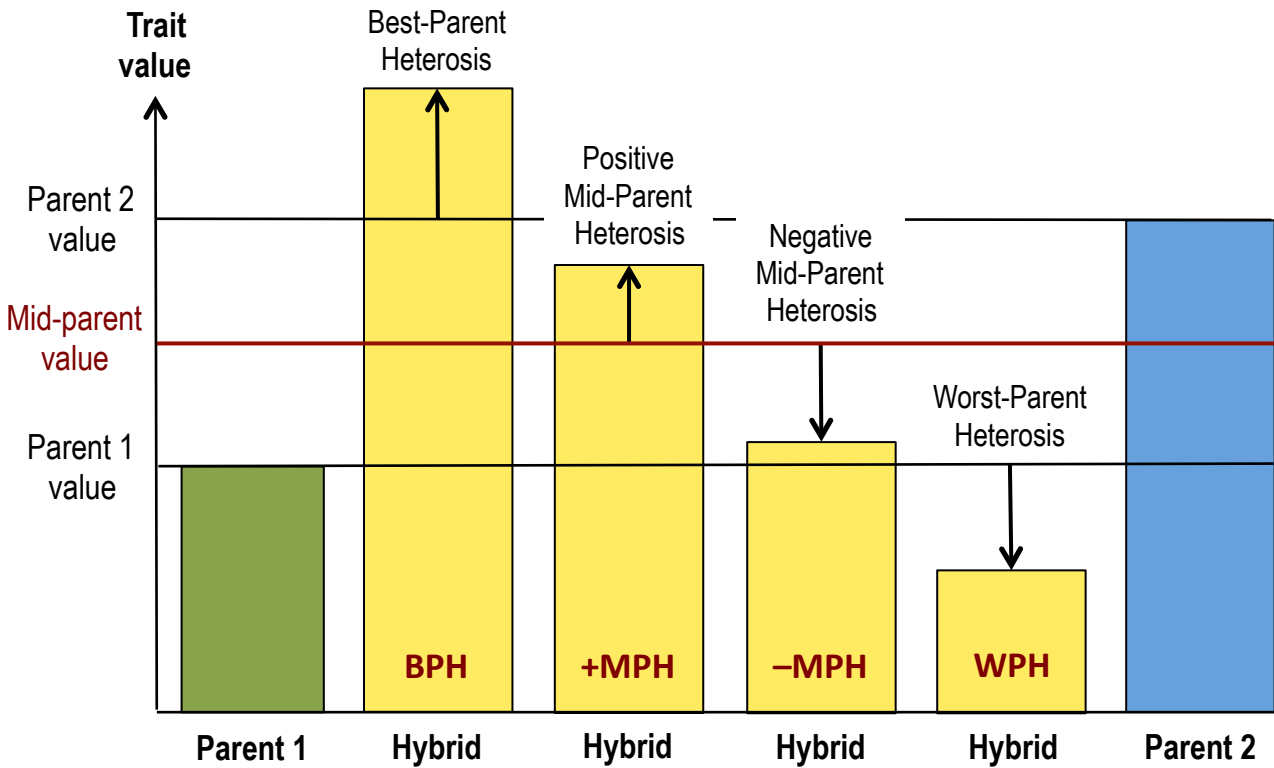

| Heterosis index | BPH      | +MPH                | -MPH                 | WPH      |
|-----------------|----------|---------------------|----------------------|----------|
| $H_{PR}$        | $> 1$    | $0 < H_{PR} \leq 1$ | $-1 \leq H_{PR} < 0$ | $< -1$   |
| $H_{BP}$        | $> 0$    | $\leq 0$            | $\leq 0$             | $\leq 0$ |
| $H_{WP}$        | $\geq 0$ | $\geq 0$            | $\geq 0$             | $< 0$    |
| $H_{MP}$        | $> 0$    | $> 0$               | $< 0$                | $< 0$    |

Supplement: Supplementary file 1 [file Data_Sheet_1.zip › Heterosis_types_V2.pdf]

A

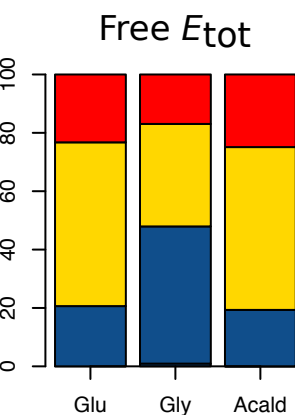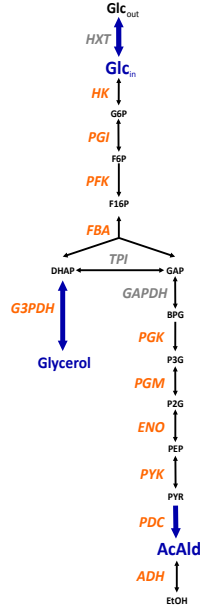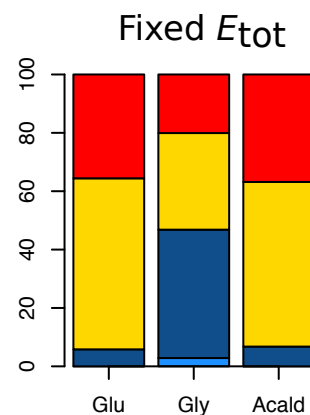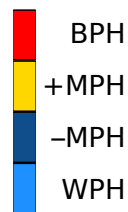

B

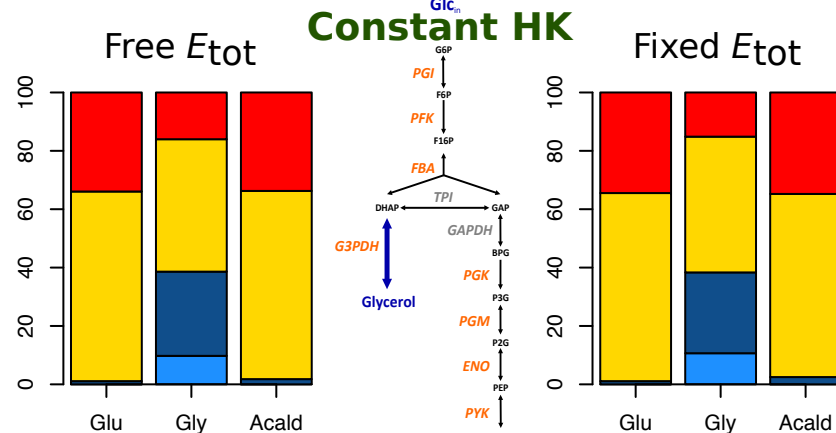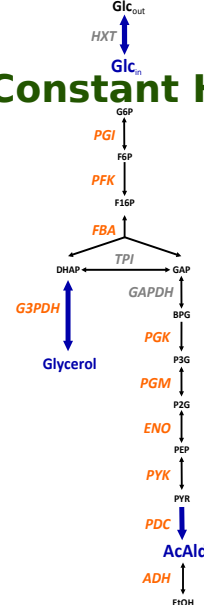

C

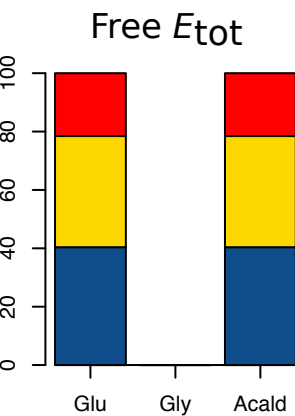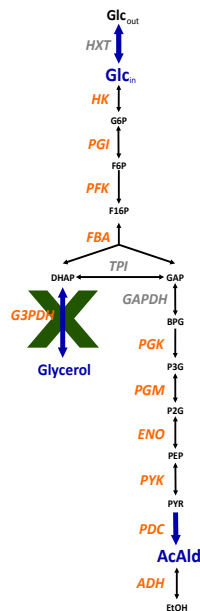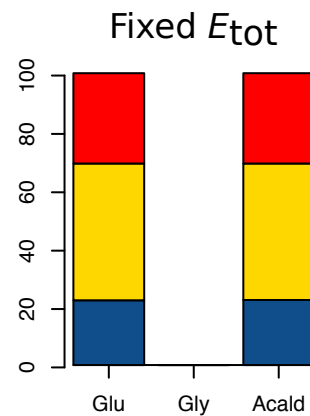

D

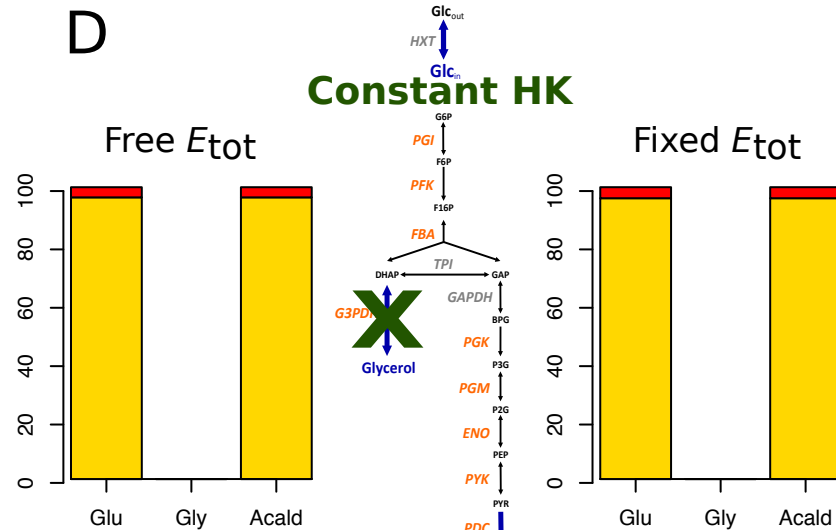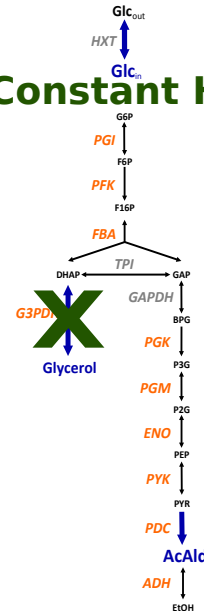

Supplement: Supplementary file 1 [file Data_Sheet_1.zip › Inheritance_concave_convex.pdf]

Free Etot

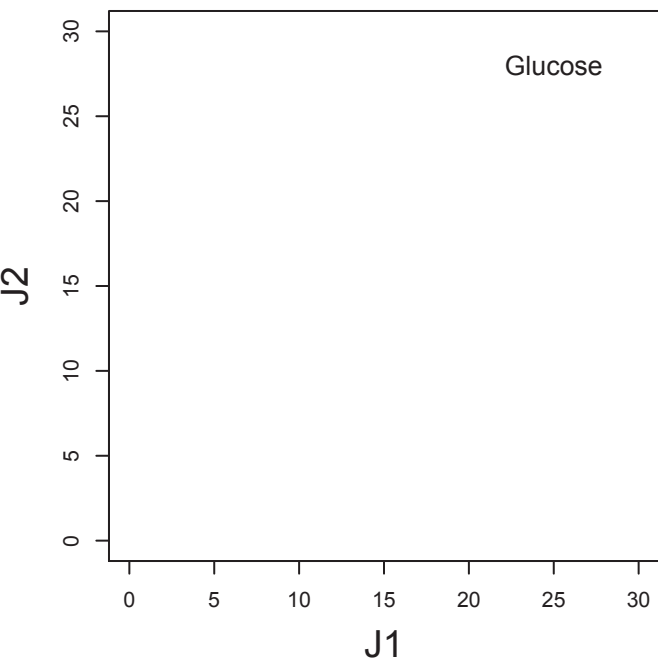

Fixed Etot

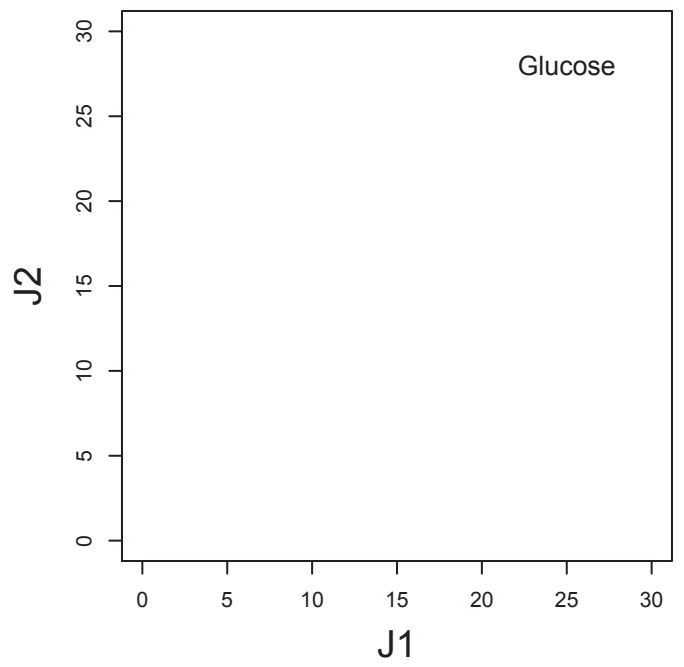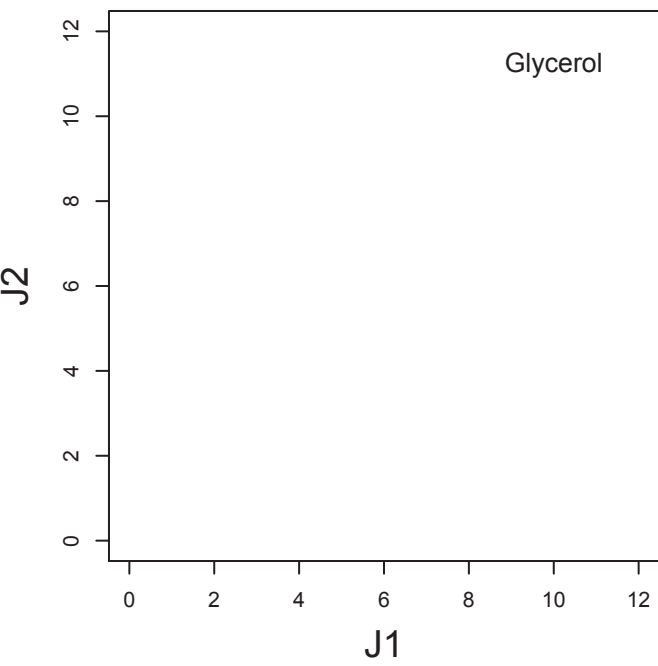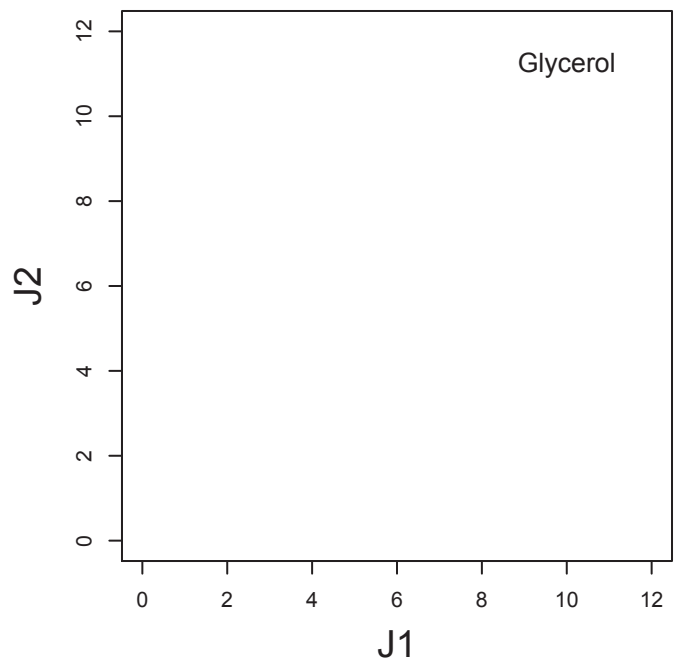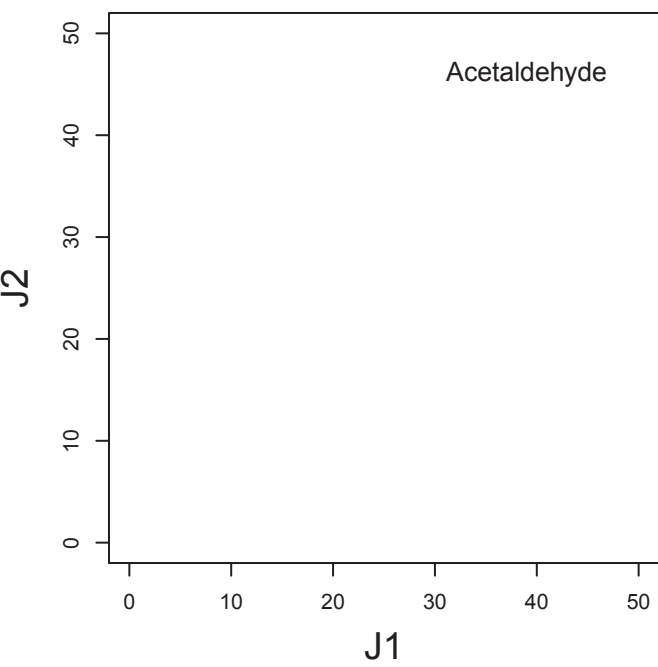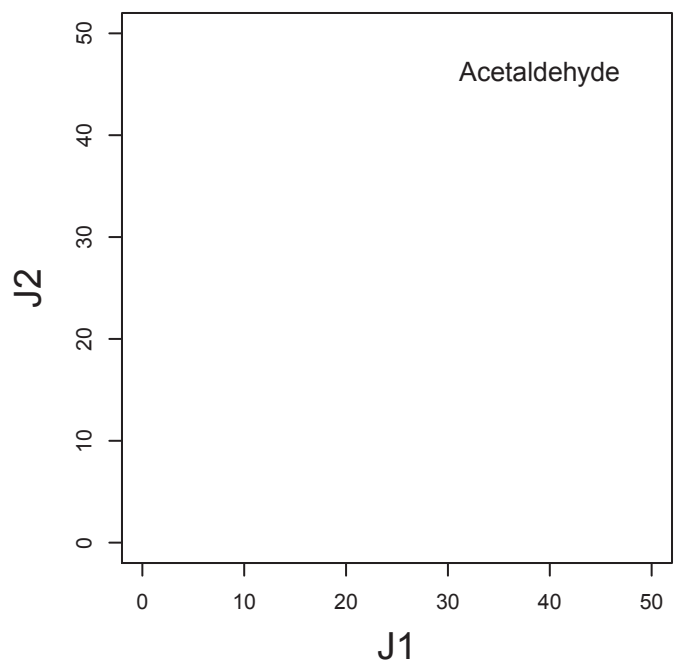

Supplement: Supplementary file 1 [file Data_Sheet_1.zip › J1-J2_Heterosis.pdf]

**A**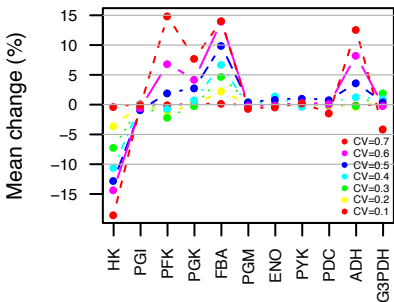**B**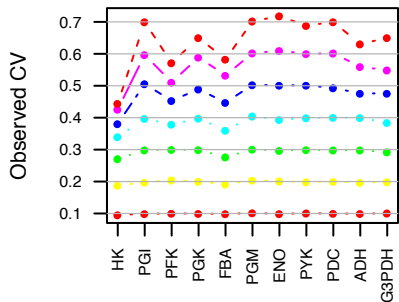**C**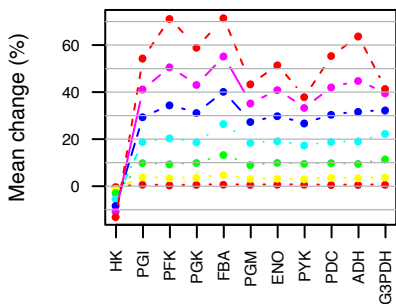**D**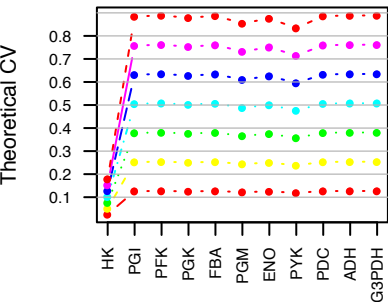**E**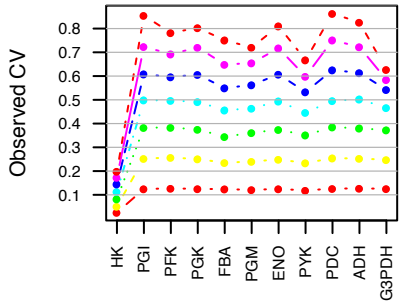

Supplement: Supplementary file 1 [file Data_Sheet_1.zip › Supp_CV_posteriori.pdf]

# Fixed Etot

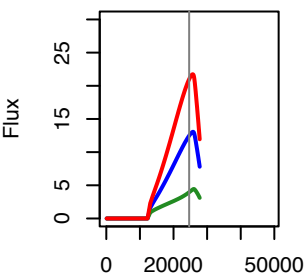

HK

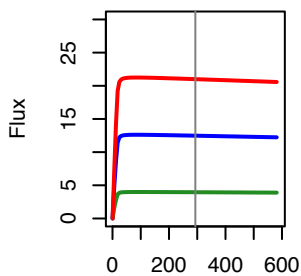

PGI

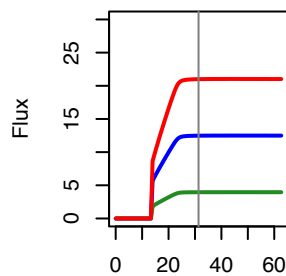

PFK

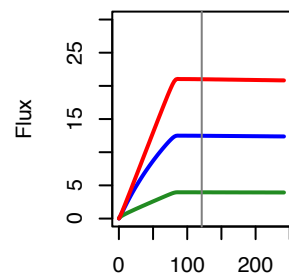

FBA

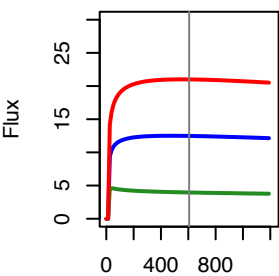

PGK

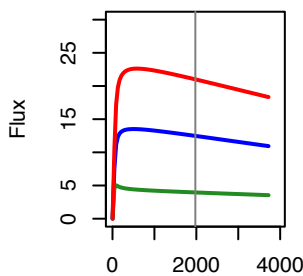

PGM

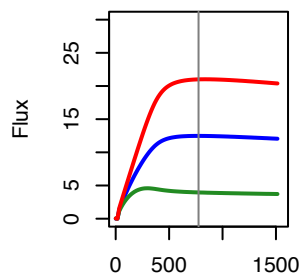

ENO

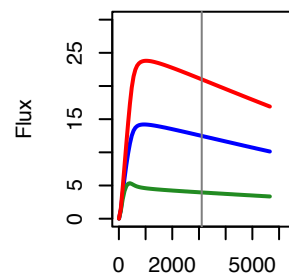

PYK

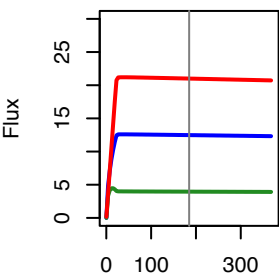

PDC

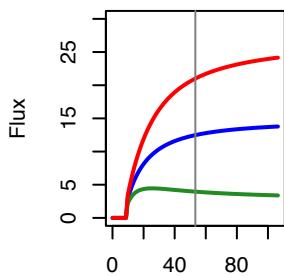

ADH

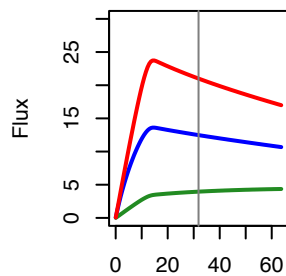

G3PDH

Acetaldehyde  
Glucose  
Glycerol

Supplement: Supplementary file 1 [file Data_Sheet_1.zip › Supp_Figure_Flux-enzymes_relationships_AC.pdf]

# Free Etot

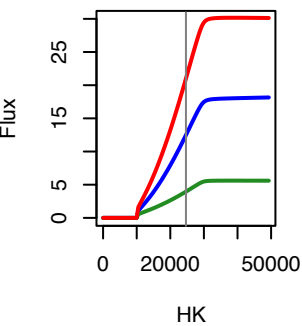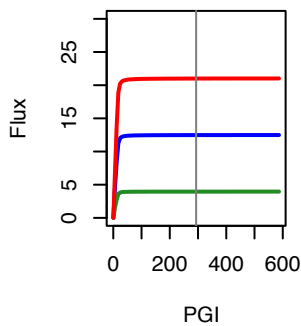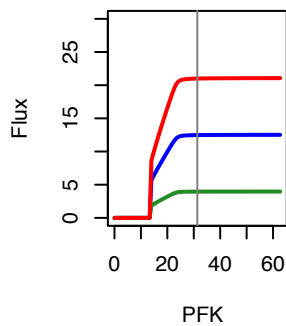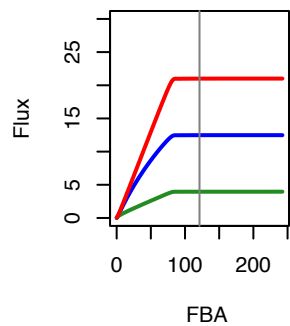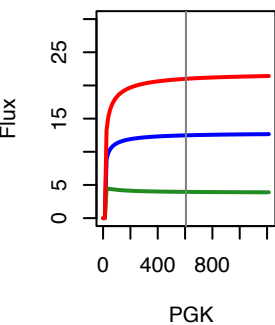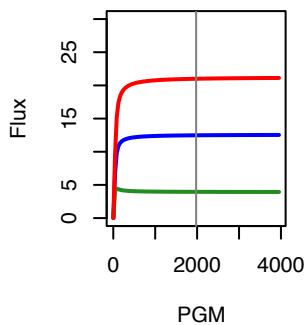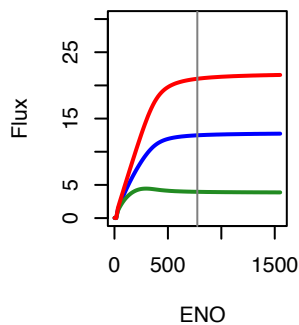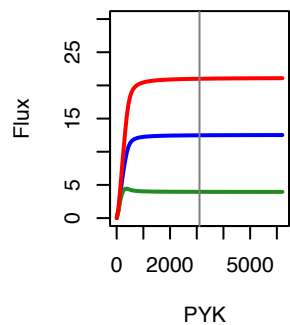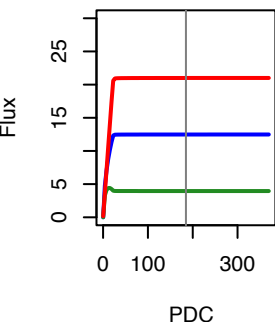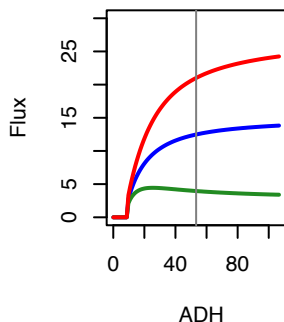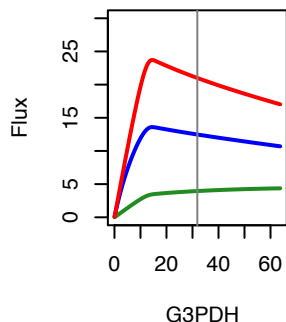

Acetaldehyde  
Glucose  
Glycerol

Supplement: Supplementary file 1 [file Data_Sheet_1.zip › Supp_Figure_Flux-enzymes_relationships_SC.pdf]

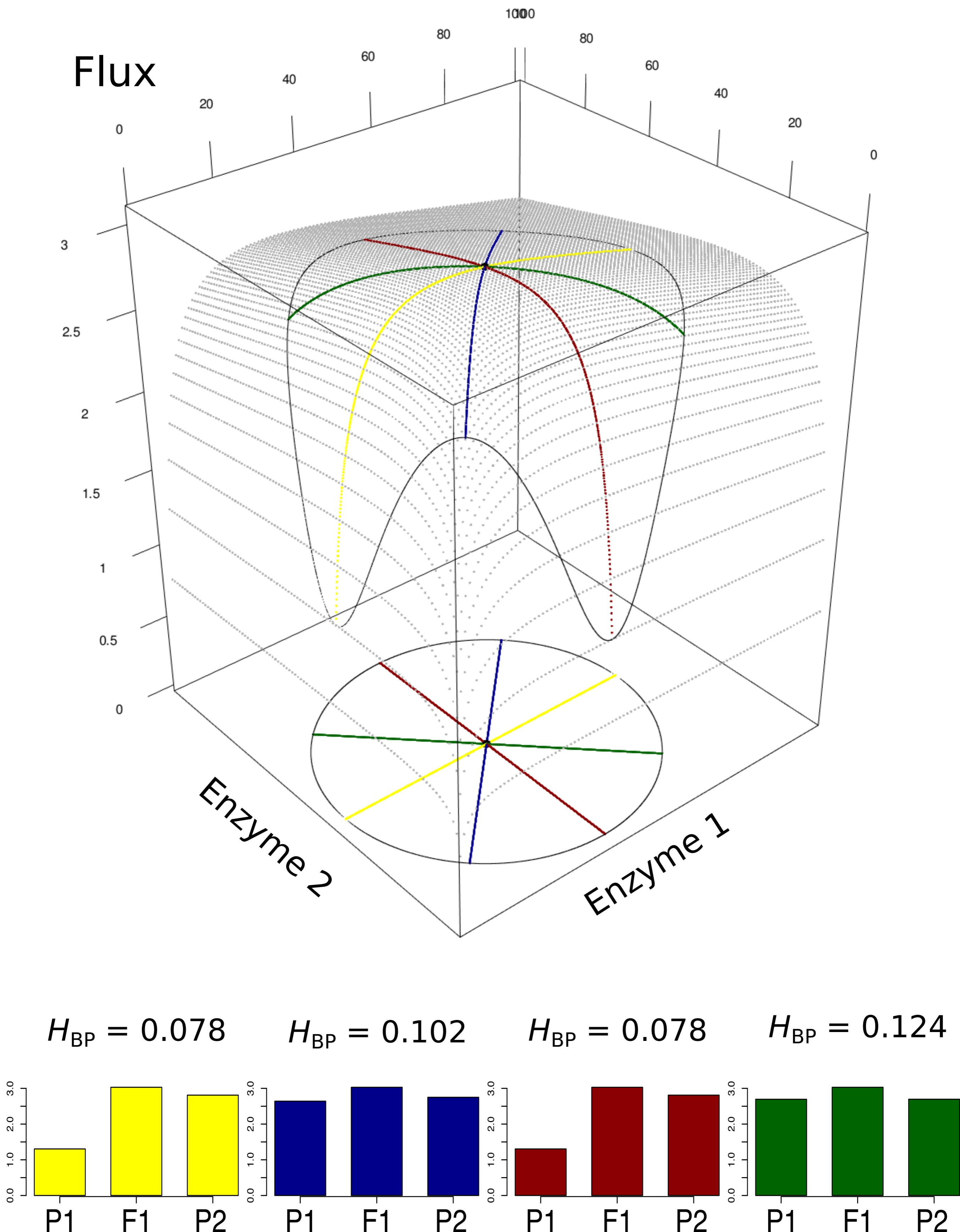

Supplement: Supplementary file 1 [file Data_Sheet_1.zip › Supp_Figure_Rotations_parents_Etot_fixe.pdf]
